# Transcription Factor ETS-1 and Reactive Oxygen Species: Role in Vascular and Renal Injury

**DOI:** 10.3390/antiox7070084

**Published:** 2018-07-03

**Authors:** Yan-Ting Shiu, Edgar A. Jaimes

**Affiliations:** 1 Division of Nephrology and Hypertension, University of Utah, Salt Lake City, UT 84112, USA; Y.Shiu@hsc.utah.edu; 2 Renal Service, Memorial Sloan Kettering Cancer Center, New York, NY 10065, USA; 3 Renal Division, Weill Cornell Medical College, New York, NY 10065, USA

**Keywords:** ETS-1, reactive oxygen species, vascular injury, renal injury

## Abstract

The E26 avian erythroblastosis virus transcription factor-1 (ETS-1) is a member of the ETS family and regulates the expression of a variety of genes including growth factors, chemokines and adhesion molecules. Although ETS-1 was discovered as an oncogene, several lines of research show that it is up-regulated by angiotensin II (Ang II) both in the vasculature and the glomerulus. While reactive oxygen species (ROS) are required for Ang II-induced ETS-1 expression, ETS-1 also regulates the expression of p47*phox*, which is one of the subunits of nicotinamide adenine dinucleotide phosphate (NADPH) oxidase and a major source of ROS in the kidney and vasculature. Thus, there appears to be a positive feedback between ETS-1 and ROS. ETS-1 is also upregulated in the kidneys of rats with salt-sensitive hypertension and plays a major role in the development of end-organ injury in this animal model. Activation of the renin angiotensin system is required for the increased ETS-1 expression in these rats, and blockade of ETS-1 or haplodeficiency reduces the severity of kidney injury in these rats. In summary, ETS-1 plays a major role in the development of vascular and renal injury and is a potential target for the development of novel therapeutic strategies to ameliorate end-organ injury in hypertension.

## 1. Introduction

Originally discovered as an oncogene, ETS-1 is now known to regulate several important biological processes in various normal cell types beyond tumors, including the regulation of immune cell functions. ETS-1 is the founding member of the ETS family, the members of which share a unique DNA binding domain called the ETS domain. The name “ETS” originates from a sequence that was discovered in 1983 in the E26 avian erythroblastosis virus, where it forms a transforming gene together with *Δgag* and *c-myb*, which encodes a 135-kDa gag-myb-ets fusion protein [1,2,3]. This newly discovered sequence was called the E26 transformation-specific sequence. In human, currently the ETS family consists of 28 members, which are classified into 4 distinct classes based on the in vitro derived binding site (Class I, II, III, IV) and 12 subfamilies based on ETS domain sequence homology (ETS, ERG, PEA3, ETV, TCF, GABP, ELF1, SPI1, TEL, ERF, SPDEF, and ESE) [4,5]. ETS-1 is the first discovered in the ETS family and belongs to Class I and the ETS subfamily.

**Figure 1:**
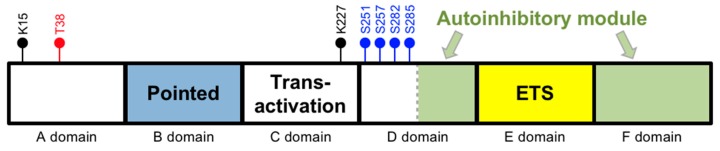
Domains of the most abundant isoform of ETS-1. Black circles are the lysine (K) sumoylation sites. Blue circles are the Ca^2+^-dependent serine (S) phosphorylation sites. The red circle is the Ras-dependent threonine (T) phosphorylation site. Note that the schematic domain lengths are not proportional to the numbers of amino acids within each domain.

In humans, the ETS-1 gene is located on chromosome 11 (11q24.3). The human TATA-less ETS-1 gene contains 8 exons, designated as exon A (first exon) followed by exons III–IX (last seven exons) [6]. Its product is the major isoform of ETS-1, which has 441 amino acids and is referred to as p54. The p54 isoform can be divided into 6 domains (Figure 1) (adapted from [7,8]). The A and B domains are regulatory units. In particular, the B domain (between amino acids 54 and 135) is the “pointed domain”, which is characterized by a sterile alpha motif (SAM) and is the second most common domain present in ETS proteins. The C domain (between amino acids 135 and 243) is the transactivation domain and contains a high content of acidic residues. The D domain (between amino acids 243 and 331) comprises two regulatory units. While its *N*-terminal unit contains several calcium-responsive phosphorylation sites, its C-terminal unit is part of an autoinhibitory module that also includes the F domain. The key functional unit of this autoinhibitory module has three inhibitory helices, which are HI-1 and HI-2 in the D domain and H4 in the F domain. The E domain (between amino acids 331 and 415) is the DNA binding ETS domain and is present in all ETS proteins. It is composed of 85 amino acids and characterized by a winged helix-turn-helix (wHTH) motif that specifically recognizes DNA sequences containing a GGAA/T core element. This domain comprises three α-helices and four β-strands that are arranged as H1-S1-S2-H2-H3-S3-S4. The autoinhibitory module’s helices block the ETS domain’s DNA binding activity by interacting with the H1-helix within the ETS domain, rendering the ETS domain in a closed confirmation and cannot bind DNA.

## 2. Regulation

The transcriptional activity of ETS-1 can be modulated through posttranslational modifications (including phosphorylation, ubiquitinylation, acetylation and sumoylation), other transcription factors, and its nuclear transportation.

### 2.1. Posttranslational Modifications

Phosphorylation of threonine 38 (Thr38) in the A domain by the mitogen-activated kinases extracellular signal regulated kinases 1 and 2 (ERK1/2) in the Ras-Raf-MAPK pathway increases the transcriptional activity of ETS-1 to promote DNA transcription [9,10,11]. The adjacent B domain (the pointed domain) serves as a docking site for ERK2. Phosphorylation of ETS-1 at Thr38 enhances the association of ETS-1 with two transcriptional co-activating proteins p300 and cAMP response element-binding protein (CREB) binding protein (CBP), resulting in an increase in the transactivational activity of their target genes [12,13]. The C-domain (the transactivation domain) is necessary for the interaction of ETS-1 with CBP/p300. Finally, it has been shown that in response to transforming growth factor (TGF)-β signaling, ETS-1 becomes acetylated and dissociated from the CBP/p300 complexes [14].

On the other hand, phosphorylation of serine residues in the D domain by calmodulin-dependent kinase II (CaMKII) inhibits DNA binding [15]. This inhibition is mediated by two mechanisms. First, serine phosphorylation in the D domain’s *N*-terminal unit increases the inhibitory effect of its C-terminal unit by stabilizing the inhibitory structure mentioned above. Second, serine phosphorylation of ETS-1 enhances its association with an E3 ubiquitin ligase, the ring finger and WD repeat domain 2 protein (RFWD2), which leads to its ubiquitination and subsequent degradation through the ubiquitin–proteasome pathway [16,17]. The exact lysine residue(s) of ETS-1 involved in ubiquitination are not yet known. Finally, mutation of the sumoylation sites in ETS-1 (K15 and K227) lead to increased transcriptional activity by ETS-1, suggesting that sumoylation of ETS-1 inhibits in its activity in transactivating genes [17,18].

### 2.2. By Transcription Factors

ETS-1 activity is further regulated through direct interactions with other transcription factors. As mentioned above, the autoinhibitory module binds to the ETS domain and the inhibitory structure is reinforced by serine phosphorylation. However, the autoinhibitory module of ETS-1 has been shown to interact with runt-related transcription factor 1 (Runx1, also termed acute myelogenous leukemia 1 (AML1) or core-binding factor α2 (CBFα2)), paired box protein Pax-5 (Pax5), transcription factor E3 (TFE3), and upstream stimulatory factor 1 (USF 1) to relieve autoinhibition [19,20,21,22,23,24]. It is important to note that, when two ETS DNA binding sites are present in the correct orientation and spacing, the autoinhibitory module of an ETS-1 protein can interact with the adjacent ETS-1 to relieve autoinhibitory function [25].

### 2.3. By Nuclear Transport

Another important mechanism by which the function of ETS proteins can be regulated is through nuclear transport. A specific region in the ETS-1 protein (between amino acids 369 and 388) called the nuclear localization sequence facilitates its movement from the cytoplasm into the nucleus, which is required for its function as a transcription factor [26].

### 2.4. Regulation of Its Expression

The expression of ETS-1 is affected by a wide spectrum of factors. Here we focus on angiotensin II (Ang II), which is a critical mediator of renal injury and vascular inflammation. In the context of kidney fibrosis, we have demonstrated that Ang II-induced expression of ETS-1 in mesangial cells was mediated by ROS [27]. In the vasculature, Ang II induced the expression of ETS-1 in cultured rat aortic smooth muscle cells, which was mediated by the G_q/11_-Src-Ras pathway [28]. More about the induction of ETS-1 by Ang II and the functional consequences are presented below. 

## 3. Effects of Angiotensin II on ETS-1 Expression and the Functional Consequences in the Vasculature and Kidney

### 3.1. In Vitro Studies

**Figure 2:**
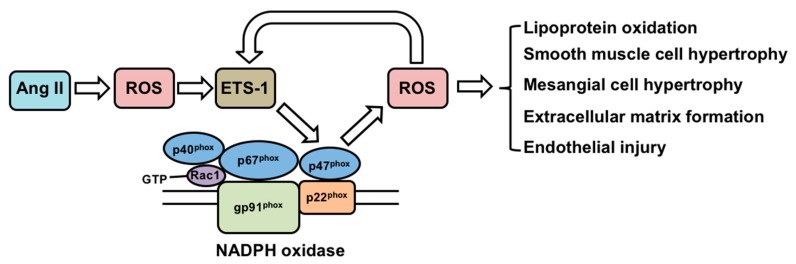
Regulation of ETS-1 by angiotensin II and the functional consequences. Ang II: Angiotensin II; ROS: reactive oxygen species; NADPH: nicotinamide adenine dinucleotide phosphate.

Angiotensin (Ang) II is a well-known mediator in the development of hypertension, atherosclerosis and renal injury [29]. Multiple studies support a role for NADPH oxidase-derived reactive oxygen species (ROS) as intermediaries of several effects of Ang II [30], including promoting lipoprotein oxidation, vascular smooth muscle hypertrophy, mesangial cell hypertrophy, extracellular matrix formation and endothelial injury [31,32] (Figure 2).

In vascular smooth muscle cells, ETS-1 is induced in response to a variety of inflammatory stimuli, including Ang II [33,34,35]. Furthermore, Ang-II induced production of the chemokine monocyte chemoattractant protein-1, the vascular cell adhesion molecule-1, and plasminogen activator inhibitor-1 in the vascular wall cells was mediated by ETS-1 [36]. In mesangial cells, Ang II increases the expression of ETS-1 via increased generation of ROS. In these cells, ETS-1 activates the production of fibronectin by directly binding an activating its promoter [37]. Thus, Ang-II induced ETS-1 in vascular smooth muscle cells and mesangial cells are of functional consequences.

In vitro studies have also demonstrated that ETS-1 is a downstream transcriptional effector of ROS. In several ovarian cancer cell lines, the expression of ETS-1 is induced by H_2_O_2_ [38]. In these cells, the induction of ETS-1 is associated with binding of the transcription factor Nrf2 to an antioxidant response element within the *ETS-1* gene promoter. ETS-1 can also collaborate with the transcription factor activator protein-1 to regulate gene expression of the matrix metalloproteinase-1 in response to H_2_O_2_ in a fibrosarcoma cell line [39]. On the other hand, ETS-1 regulates the expression of p47*phox*, which is one of the subunits of NADPH oxidase and a major source of ROS in the kidney and vasculature [40]. Taken together, these studies support a dual role of ETS-1 as a downstream mediator of ROS, as well as an upstream regulator of ROS via regulating the expression of genes that are involved in the ROS generation (Figure 2).

As we and others have shown, the production of ROS is a critical determinant in Ang II-induced vascular remodeling that is independent of changes in blood pressure [32,41,42]. ROS also play a major role as mediators of extracellular matrix deposition, mesangial cell proliferation and hypertrophy in the kidney in response to Ang II [32]. In glomerular mesangial cells, we have shown that Ang II-induced ETS-1 expression is prevented by ROS inhibition, suggesting that ROS are necessary for ETS-1 expression [27]. We also showed that ETS-1 is one of the transcription factors that transactivate the expression of fibronectin induced by Ang II. This transactivation occurs through the interaction of ETS-1 with CREB and p300. In addition, we showed that the phosphorylation of ETS-1 induced by Ang II involves both ERK-MAPK and PI3K/Akt pathways [37].

### 3.2. In Vivo Studies

The renal expression of ETS-1 is augmented in a variety of models of renal injury. In the anti-Thy model of glomerulonephritis, the expression of ETS-1 is increased in the glomerular mesangium and to a lesser degree in podocytes and glomerular endothelium [43]. In a model of acute renal failure, the tubular expression of ETS-1 is increased and associated with increased expression of cyclin D, suggesting that ETS-1 participates in the control of tubular regeneration in acute renal failure [44]. In rats with anti-glomerular basement induced glomerulonephritis, there is also increased expression of ETS-1 in the glomeruli and in the interstitium [45].

**Figure 3:**
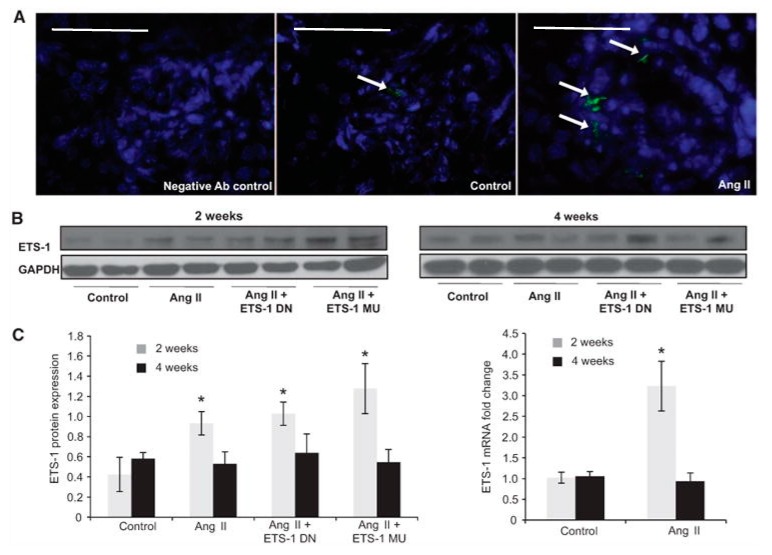
Angiotensin II (Ang II) increases cortical ETS-1 expression. (**A**) Representative confocal photomicrographs showing low basal expression of ETS-1 (green) in control kidney cortex, which is predominantly glomerular and increased by Ang II. (Bar = 50 μm); (**B**) The renal cortical ETS-1 protein expression increases after 2 weeks of Ang II as assessed by Western blot (*n* = 6, * *p* < 0.05 vs. control) and returns to baseline after 4 weeks of Ang II. The expression of ETS-1 was not significantly modified by treatment with either ETS-1 dominant-negative (ETS-1 DN) or ETS-1 mutant (ETS-1 MU) peptide; (**C**) Densitometric analysis for ETS-1 showing significant increases in ETS-1 protein expression after 2 but not after 4 weeks of Ang II. Neither ETS-1 DN nor ETS-1 MU modified ETS-1 protein expression. Data expressed as mean ± SEM are normalized to GAPDH (* *p* < 0.05 vs. control; *n* = 6). The infusion of Ang II for 2 weeks resulted in increases in cortical ETS-1 mRNA expression as assessed by real-time reverse transcriptase polymerase chain reaction (*n* = 6; * *p* < 0.05 vs. control) and returns to baseline levels after 4 weeks of Ang II. GAPDH: glyceraldehyde 3-phosphate dehydrogenase. (Right panel).

In vivo studies have also determined that Ang II increases renal ETS-1 expression. We have found that the infusion of Ang II results in a 3-fold increase in ETS-1 mRNA expression and a 4-fold increase in ETS-1 protein expression as compared with control mice [46] (Figure 3). By immunofluorescence, we determined that the increase in ETS-1 expression was mostly in the glomeruli. In these mice, blockade of ETS-1 utilizing a dominant negative peptide (ETS-DN) reduced proteinuria, mesangial expansion and expression of α-smooth muscle actin, a well-validated marker of renal fibrosis [46]. Blockade of ETS-1 also reduced the mRNA and protein expression of the growth factors TGF-β and CTGF, as well as cell proliferation and macrophage infiltration [46]. The infusion of Ang II resulted in increased expression of NOX4 and nitrotyrosine, which were also reduced by ETS blockade. These findings demonstrate that the inductive effects of Ang II on renal fibrosis, inflammation and increased oxidative stress are at least in part mediated by ETS-1. Importantly, ETS-1 blockade did not have a significant effect on blood pressure in mice infused with Ang II.

Others have demonstrated that in response to systemic Ang II infusion, ETS-1 is induced in vascular smooth muscle cells and endothelial cells of the mouse thoracic aorta [36]. Furthermore, Ang II-induced arterial wall thickening, perivascular fibrosis, cardiac hypertrophy and recruitment of T cells and macrophages to the vessel wall were significantly diminished in *ETS-1^−/−^* mice as compared with control mice. Ang II-induced cyclin-dependent kinase inhibitor p21CIP, plasminogen activator inhibitor-1, and monocyte chemoattractant protein-1 were also reduced in the aorta of ETS-1^−/−^ mice compared with wild-type controls [36].

In addition, Ang II-induced production of H_2_O_2_ and superoxide anion (O_2_^−^) was blunted in vascular smooth muscle cells from *ETS-1^−/−^* mice. In primary human aortic smooth muscle cells, Ang II-induced ROS production and the induction of the NADPH oxidase subunit p47phox were inhibited by small interfering RNA of ETS-1. In addition, blockade of ETS-1 with ETS-DN reduced Ang II–induced ROS production and medial hypertrophy in the mouse thoracic aorta [47].

## 4. ETS-1 in Neointima Formation

**Figure 4:**
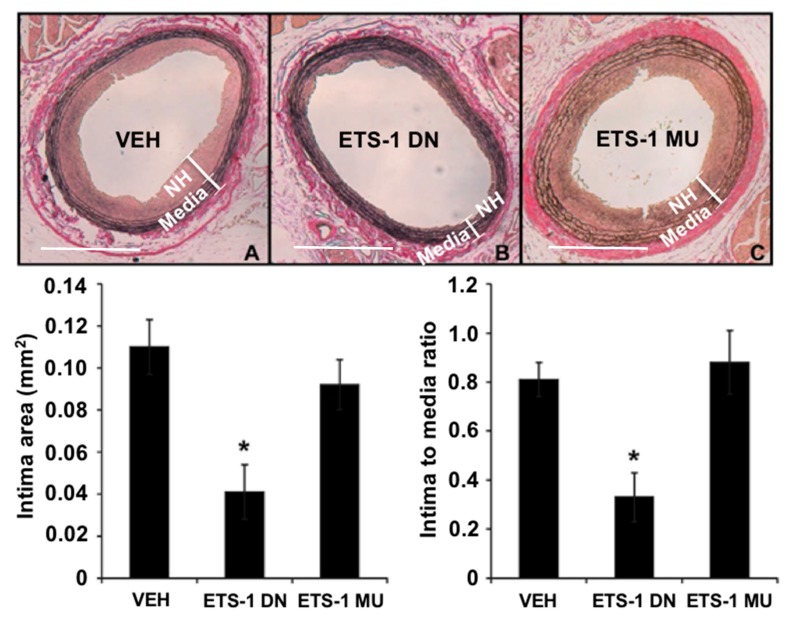
A single injection of ETS-1 DN (10 mg/kg) inhibited the formation of neointimal hyperplasia (NH) in injured rat carotid arteries. Top, elastic Van Gieson-stained tissue sections of carotid arteries from rats treated with (**A**) saline vehicle (VEH); (**B**) ETS-1 DN; or (**C**) ETS-1 mutant (MU) 14 days after balloon injury. Bottom, composite data are shown as mean ± SEM (*n* = 6 per group). * *p* < 0.05 compared to the VEH group. (Bar = 50 μm).

The formation of neointimal hyperplasia is a common response to vascular injury. It is the result of migration and proliferation of vascular smooth muscle cells into the intimal layer and is the major cause of restenosis after percutaneous coronary intervention such as balloon angioplasty and stenting [48]. Using a rat carotid artery balloon injury model, we found that mRNA expression of ETS-1 was increased rapidly (within 2 h) in injured carotid arteries and we confirmed increased nuclear expression of ETS-1 within 24 h postinjury [48]. Importantly, we found that blockade of ETS-1 with ETS-1 DN effectively inhibited neointima formation in this model (Figure 4). We also demonstrated that ETS-1 mediated the formation of arterial neointima in this model by regulating the activation of proinflammatory cytokines and adhesion molecules, including IL-6, monocyte chemoattractant protein 1 (MCP-1), P-selectin, and E-selectin [48]. Neointima formation can also occur in the vein, such as in an arteriovenous fistula (created by a direct anastomosis between an artery and a vein) or an arteriovenous graft that is used as hemodialysis vascular access. Using a mouse model of carotid-jugular fistula, we found that the mRNA expression of ETS-1 was increased in the venous limb of the fistula, and we confirmed increased expression of ETS-1 predominantly in the neointima and overlying endothelium [49]. Furthermore, blockade of ETS-1 with ETS-1 DN inhibited the formation of neointima in the mouse carotid-jugular fistula, as well as reduced the expression of nitric oxide synthase 2, NOX2, NOX4, E-selectin, and MCP-1 [49]. While we also found an increased protein and nuclear expression of ETS-1 in the neointima of a porcine carotid-jugular graft model, we did not find an increase of ETS-1 at the mRNA level [49].

## 5. ETS-1 in Salt-Sensitive Hypertension

Salt-sensitive (SS) hypertension affects over 50% of the general population [50] and is associated with a high risk for hypertensive end-organ damage including atherosclerosis, left ventricular hypertrophy and chronic kidney disease [51]. As we have previously shown, SS hypertension is characterized by reduced cardiovascular and renal NO bioavailability [51]. The renin angiotensin system (RAS) has been recognized as an endocrine, paracrine, autocrine and intracrine system [52,53,54]. Although all organs have elements of the RAS, the kidney is unique in that it has every component of the RAS [55]. Importantly the tissue concentration of Ang II in the kidneys are several fold higher than can be explained by Ang II delivery from arterial blood flow [56]. Dahl salt-sensitive rats (DS rats) are a well-established model of salt-sensitive hypertension that develop hypertension when fed a high salt diet [57]. Although these rats have low levels of circulating RAS several studies have shown improvement of cardiac and renal function when treated with an angiotensin-converting enzyme (ACE) inhibitor or angiotensin II receptor blockers (ARB) [58,59,60,61,62,63], suggesting local activation of RAS. Although these rats have suppressed plasma renin when placed on a high salt diet the kidney levels of angiotensinogen are markedly increased indicating a paradoxical augmentation of intrarenal angiotensinogen that appears to be mediated dependent on the generation of reactive oxygen species [64,65,66].

**Figure 5:**
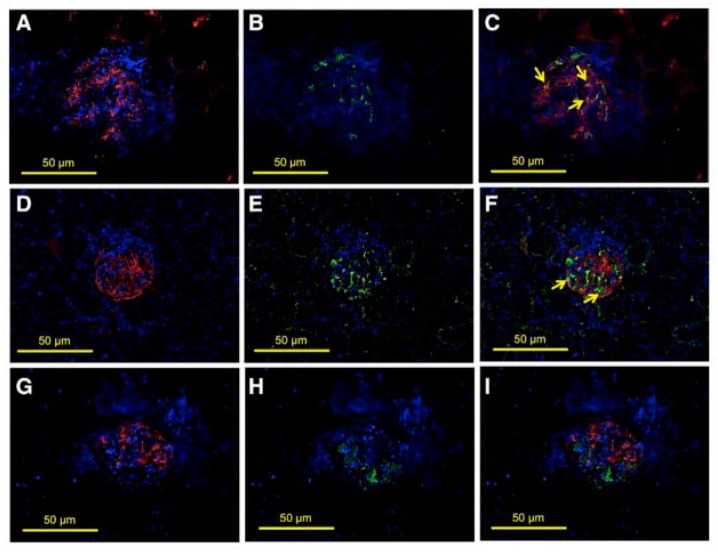
Glomerular expression of transcription factor avian erythroblastosis virus E26 oncogen homolog-1 (ETS-1) in hypertensive Dahl salt-sensitive (DS) rats. Colocalization studies were performed to characterize the expression of ETS-1 in the glomerular endothelium (CD31), podocytes (synaptopodin), or mesangium (desmin). (**A**–**C**) show positive stain for CD31 (**A**, red), ETS-1 (**B**, green), and some areas in which there is colocalization of CD31 and ETS-1 indicating endothelial expression of ETS-1 (**C**, arrows); (**D**–**F**) show positive stain for synaptopodin (**D**, red), ETS-1 (**E**, green), and areas in which there is colocalization of ETS-1 and synaptopodin indicating expression of ETS-1 in podocytes (**F**, arrows); (**G**–**I**) show positive stain for desmin (**G**, red), ETS-1 (**H**, green); no clear evidence of colocalization of ETS-1 and desmin was observed (**I**), suggesting lack of expression of ETS-1 in mesangial areas (×40 magnification).

We have demonstrated increased glomerular expression of ETS-1 in hypertensive Dahl salt-sensitive rats [67]. These rats had a significant increase in the phosphorylated ETS-1 level without a change in the total ETS-1 level. Phosphorylated ETS-1 was primarily located in the glomeruli and predominantly expressed in the glomerular epithelium and to a lesser degree in the glomerular endothelium (Figure 5).

Studies have demonstrated that Dahl salt-sensitive rats, when fed a high salt diet and made hypertensive, have increased local activation of the RAS, characterized by sustained levels of Ang II, increased levels of angiotensinogen and increased expression of the AT1 receptor [64]. In addition, as others and we have shown, salt-sensitive hypertension is associated with reduced NO bioavailability and increased ROS production [51,68,69,70]. In support of the role for increased RAS activation in salt-sensitive hypertension, AT1 receptor blockade ameliorates cardiac and/or renal dysfunction in these rats, suggesting an important role for RAS in the development of end-organ injury in salt-sensitive hypertension [65,66]. We observed an increased level of phosphorylated ETS-1 in hypertensive DS rats, and this increase was significantly reduced by RAS blockade with ARB, suggesting that increased RAS activation mediates increased ETS-1 phosphorylation in hypertensive DS rats [67]. To determine the role of ETS-1 in the pathogenesis of renal injury in salt-sensitive hypertension, we used a dominant negative ETS-1 peptide that competes for DNA binding with ETS-1 but does not initiate gene transcription. We observed that ETS-1 blockade reduced ETS-1 phosphorylation, which is consistent with a positive feedback of ETS-1 on its own activation.

ETS-1 blockade alone resulted in significant reductions in glomerular injury score, fibronectin expression, proteinuria and macrophage infiltration but had no significant effect on interstitial fibrosis. RAS blockade alone also reduced glomerular injury score, proteinuria and macrophage infiltration but had no significant effect on fibronectin production or fibrosis. In contrast, concomitant ETS-1 and RAS blockade had additive effects on all parameters examined. In addition, we observed that ETS-1 blockade resulted in a significant reduction in the urinary excretion of TGF-β, suggesting that ETS-1 may be a direct regulator of TGF-β in hypertensive DS rats. In contrast, RAS blockade did not modify the urinary excretion of TGF-β, indicating that other pathways independent of RAS participate in the production of TGF-β in hypertensive DS rats. Both ETS-1 blockade alone and RAS blockade alone had very small effects on blood pressure as measured by radio-telemetry. However, rats with combined ETS-1 and RAS blockade had blood pressures that were similar to those from rats on a normal salt diet, indicating that ETS-1 may also be playing a role in blood pressure regulation either directly or indirectly. These findings also suggest that the additional beneficial effects of concomitant ETS-1 and RAS blockade are in large part due to their effects on blood pressure [67].

To better understand the interaction between RAS and ETS-1, we measured the expression of some of main components of RAS in the different experimental groups. We and others have previously shown that hypertensive DS rats had significant increases in the urinary excretion of angiotensinogen and intrarenal concentration of Ang II, which are indicative of increased RAS activation. Both ETS-1 blockade alone as well as RAS blockade alone induced significant reductions in the urinary excretion of angiotensinogen and tissue levels of Ang II. Concomitant ETS-1 and RAS blockade further reduced the urinary excretion of angiotensinogen. These findings suggest that ETS-1 also plays a role in the regulation of the RAS, through the mechanisms are unclear.

In our recent studies, when placed on high salt diet, DS rats with only one functioning ETS-1 gene had lower glomerular injury score, less albuminuria, lower serum creatinine and less kidney fibrosis when compared to wild type controls [71]. Moreover, transplant of a kidney from rats with only one copy of the ETS-1 gene into salt resistant rats (DR) resulted in less glomerular injury and proteinuria as compared to rats transplanted with a kidney receiving a transplant from a DS rat. These results demonstrate that reduced renal expression of ETS-1 prevents hypertension-associated kidney injury in SS rats [71].

## 6. Conclusions

In conclusion, multiple sources of experimental evidence demonstrate a role of ETS-1 in vascular and renal injury. ETS-1 is upregulated by Ang II and regulates the expression of cytokines, growth factors and adhesion molecules. Reactive oxygen species are required for ETS-1 expression in response to Ang II but at the same time ETS-1 regulates the activity of NADPH oxidase in the vasculature. ETS-1 is upregulated the vasculature following injury and drives the formation of neointima. ETS-1 is also upregulated in salt-sensitive hypertension, where it plays an important role in the development of end-organ injury. The current evidence suggests that ETS-1 could be a potential therapeutic target for the treatment or prevention of end-organ injury in hypertension since beneficial RAS and ETS-1 blockade have additive beneficial effects on the severity of injury suggesting that ETS-1 can also be upregulated independently of RAS activation.
